# Low HIV incidence in pregnant and postpartum women receiving a community-based combination HIV prevention intervention in a high HIV incidence setting in South Africa

**DOI:** 10.1371/journal.pone.0181691

**Published:** 2017-07-27

**Authors:** Geoffrey Fatti, Najma Shaikh, Debra Jackson, Ameena Goga, Jean B. Nachega, Brian Eley, Ashraf Grimwood

**Affiliations:** 1 Kheth’Impilo, Cape Town, South Africa; 2 South African Centre for Epidemiological Modelling and Analysis (SACEMA), Stellenbosch University, Stellenbosch, South Africa; 3 UNICEF, New York, New York, United States of America; 4 School of Public Health, University of the Western Cape, Cape Town, South Africa; 5 Health Systems Research Unit, South African Medical Research Council, Pretoria, South Africa; 6 Department of Paediatrics, University of Pretoria, Pretoria, South Africa; 7 Departments of Epidemiology, Infectious Diseases and Microbiology, University of Pittsburgh Graduate School of Public Health, Pittsburgh, Pennsylvania, United States of America; 8 Department of Medicine and Centre for Infectious Diseases, Faculty of Medicine and Health Sciences, Stellenbosch University, Cape Town, South Africa; 9 Departments of Epidemiology and International Health, Johns Hopkins University Bloomberg School of Public Health, Baltimore, Maryland, United States of America; 10 Department of Paediatrics and Child Health, Red Cross War Memorial Children’s Hospital, University of Cape Town, Cape Town, South Africa; National and Kapodistrian University of Athens, GREECE

## Abstract

**Background:**

Young Southern African women have the highest HIV incidence globally. Pregnancy doubles the risk of HIV acquisition further, and maternal HIV acquisition contributes significantly to the paediatric HIV burden. Little data on combination HIV prevention interventions during pregnancy and lactation are available. We measured HIV incidence amongst pregnant and postpartum women receiving a community-based combination HIV prevention intervention in a high HIV incidence setting in South Africa.

**Methods:**

A cohort study that included HIV-uninfected pregnant women was performed. Lay community-based workers provided individualized HIV prevention counselling and performed three-monthly home and clinic-based individual and couples HIV testing. Male partners were referred for circumcision, sexually transmitted infections or HIV treatment as appropriate. Kaplan-Meier analyses and Cox’s regression were used to estimate HIV incidence and factors associated with HIV acquisition.

**Results:**

The 1356 women included (median age 22.5 years) received 5289 HIV tests. Eleven new HIV infections were detected over 828.3 person-years (PY) of follow-up, with an HIV incidence rate of 1.33 infections/100 PY (95% CI: 0.74–2.40). Antenatally, the HIV incidence rate was 1.49 infections/100 PY (95% CI: 0.64–2.93) and postnatally the HIV incidence rate was 1.03 infections/100 PY (95% CI: 0.33–3.19). 53% of male partners received HIV testing and 66% of eligible partners received referral for circumcision. Women within known serodiscordant couples, and women with newly diagnosed HIV-infected partners, adjusted hazard ratio (aHR) = 32.7 (95% CI: 3.8–282.2) and aHR = 126.4 (95% CI: 33.8–472.2) had substantially increased HIV acquisition, respectively. Women with circumcised partners had a reduced risk of incident HIV infection, aHR = 0.22 (95% CI: 0.03–1.86).

**Conclusions:**

Maternal HIV incidence was substantially lower than previous regional studies. Community-based combination HIV prevention interventions may reduce high maternal HIV incidence in resource-poor settings. Expanded roll-out of home-based couples HIV testing and initiating pre-exposure prophylaxis for pregnant women within serodiscordant couples is needed in Southern Africa.

## Background

Southern Africa is the epicentre of the HIV pandemic having the highest burden of HIV globally [1]. Young women and adolescent girls are the demographic group having the highest HIV incidence, up to eight-fold higher than their male peers, attributed to complex social, behavioural, biologic and structural factors [2–4].

HIV acquisition is further increased two to four-fold during pregnancy, due to biological and behavioural factors including immunological changes, hormonal changes affecting the genital tract mucosa, higher frequency of unprotected sex and incident sexually transmitted infections (STIs) during pregnancy [5–9]. HIV incidence remains increased during the postnatal period [10,11]. In addition, mother-to-child transmission (MTCT) amongst women who acquire HIV during pregnancy/lactation is double to triple compared to women who acquire HIV prior to pregnancy [11,12], and contributes significantly to the Southern African pediatric HIV burden [13,14].

HIV prevention in young women in sub-Saharan Africa is a public health imperative [15], and prevention efforts during pregnancy and postpartum should be particularly prioritized due to the increased risk of HIV acquisition in both women and infants [10,11]. Prevention efforts have included structural, biomedical and behavioural interventions [16]. Behavioural interventions have reported modest impact on self-reported HIV-preventive behaviours but little impact on HIV incidence [3,17]. Biomedical prevention for pregnant and breastfeeding women in South Africa is limited due to oral pre-exposure prophylaxis (PrEP) being contraindicated in current guidelines because of limited safety data regarding the developing fetus and infant [18,19], despite the World Health Organization’s (WHO) guidelines indicating that PrEP “can be used during pregnancy” [20]. As HIV epidemics are complex due to a variety of contextual risk factors, programs that incorporate comprehensive combination interventions are important [3,4].

Health systems interventions involving community-based workers have become increasingly important in sub-Saharan Africa due to severe professional health worker shortages and the WHOs recommendations regarding task-shifting [21,22]. Lay health worker interventions have shown successes in HIV treatment programs [23,24], and the South African Department of Health’s strategy to re-engineer primary healthcare emphasizes the critical role of community health workers [25].

An important current knowledge gap is the lack of data regarding the effectiveness of combination HIV prevention interventions during pregnancy and postpartum [4,26]. Evaluating community-level interventions for maternal and neonatal health is also a current research priority [27].

A combination HIV prevention intervention for pregnant and postpartum women utilizing community-based health workers has recently been established in KwaZulu-Natal province, South Africa, a region having high HIV incidence [6,28]. This study aimed to measure this interventions effectiveness in preventing incident HIV infection in this vulnerable key population, and to investigate factors associated with HIV acquisition, and were aims that were achieved.

## Methods

### Study design, setting and inclusion criteria

A cohort study utilizing enhanced routine clinical data was performed at an urban primary healthcare facility north of Durban in eThekwini district. The district antenatal HIV prevalence in 2013 was 41.1% having increased from 38.0% in 2011 [29], with HIV incidence in nonpregnant women being 6.4 infections/100 person-years (PY) [28].

All pregnant women who presented for antenatal care between 01 March 2013 and 31 May 2015 who tested HIV-negative at the first antenatal visit, who gave informed consent to enrol in the community-based program, and who had one or more follow-up HIV tests at least 8 weeks after the initial HIV test were included in analyses. Women were followed-up with their infants until the earliest of 18 months postpartum, transfer to another clinic, loss to follow-up (LTFU), mortality, or 28 February 2016. For HIV-infected pregnant and breastfeeding women, national guidelines recommended WHO option B (immediate triple ART until the end of breastfeeding or lifelong if CD4 cell count < 350 cells/μL) prior to January 2015 [30], and lifelong ART irrespective of CD4 cell count thereafter [31].

### Community-based combination HIV prevention intervention

The intervention aimed to reduce HIV incidence in pregnancy and postpartum, thus to reduce the number of infants being HIV-exposed. Behavioural and psychosocial component interventions included individual counselling and education for women and their male household partners regarding HIV risk and safe sex practices (including promotion of condom use), education regarding multiple and concurrent sexual partnerships, couples counselling, counselling regarding HIV serodiscordant couples, addressing alcohol and substance abuse, assessing and addressing mental health needs, and group HIV prevention education at the clinic. Biomedical interventions involved provision of male and female condoms, home and clinic-based three-monthly individual and couples HIV testing and counselling (HTC) by community workers throughout pregnancy until 18 months postpartum, facilitated linkage to HIV care facilities for either partner testing HIV positive with subsequent antiretroviral treatment (ART) adherence counselling, referral of eligible men for voluntary male medical circumcision (VMMC), and symptom screening of women and male partners for sexually transmitted infections (STIs) with referral for treatment if symptomatic. A structural component of the intervention involved assessment and counselling and referral for gender-based violence as required, and discussion regarding gender identities and roles.

All women were assigned a community-based healthcare worker, named a Patient Advocate (PA), who provided home and clinic-based support two to three-weekly until six weeks postpartum, and monthly thereafter. PAs also provided education regarding contraception and reproductive health, infant health and feeding practices, encouraged women to attend all antenatal and infant immunization clinic visits, and supported women to access government social security grants. Non-household male partners were invited to receive HTC at the facility through word of mouth messages, invitation cards and telephonic invitations, but were not physically traced to their homes.

Disclosure of HIV serostatus was addressed by PAs at enrolment and during HIV test counselling. HIV test results were kept confidential by PAs (including in the case of minors with regards to caregivers). PAs discussed possible disclosure of positive HIV status of participants to others (including partners and caregivers); however, the decision whether to disclose HIV status resided with the participant. An anonymized key was used to identify study participants for data analyses.

The intervention was instituted by Kheth’Impilo, a nonprofit organization supporting the Department of Health with public health systems innovations. PAs originally provided community-based adherence support to ART patients [24,32], and have more recently been utilized for HIV prevention.

### Outcomes and definitions

The primary outcome was the HIV incidence rate and cumulative HIV incidence amongst HIV-uninfected pregnant and postpartum women. Secondary outcomes (other program effectiveness and process measures) were: I) HIV incidence during pregnancy; II) Postpartum HIV incidence (until 18 months); III) Socio-demographic factors associated with incident HIV infection; IV) MTCT at 6 weeks postpartum amongst women with incident HIV infection prior to 6 weeks postpartum (proportion of HIV-tested infants testing HIV positive); V) Time from diagnosis until ART initiation amongst women with incident HIV; VI) Proportion of women with incident antenatal HIV infection who initiated ART antenatally; VII) Maternal mortality rate; VIII) Cumulative probability of LTFU of enrolled women; IX) Socio-demographic factors associated with LTFU; X) Proportion of male partners who received HTC (with recorded test results); XI) Proportion of partners testing HIV positive successfully linked with HIV care facilities; XII) Time from HIV diagnosis till ART initiation amongst partners eligible to initiate ART; XIII) Proportion of eligible partners referred for VMMC.

Adult HIV testing was performed using the Advanced Quality^(TM)^ Rapid Anti-HIV (1&2) test, and positive tests were confirmed with the Abon^(TM)^ HIV 1/2/O Tri-line Rapid test device. Infant HIV testing was performed with HIV deoxyribosenucleic acid polymerase chain reaction (PCR) testing. Maternal deaths were recorded as per professional healthcare worker or PA report and defined as deaths during pregnancy or until 42 days of termination of pregnancy related to or aggravated by the pregnancy. LTFU was defined as no contact with clinic staff or PA for at least 120 days from last recorded visit, or if reported as LTFU by the PA. Men eligible to be referred for VMMC were uncircumcised males (by self-report) or who had unknown circumcision status who tested HIV negative or who had unknown HIV status.

### Data collection and statistical analyses

Enhanced routine clinical data were prospectively collected by site-based data capturers from clinical records in a custom-designed electronic database following participant clinic visits. PAs collected home visit data with paper forms. Data accuracy were reviewed by the district data co-ordinator and data queries resolved by site and community-based staff.

For HIV incidence, person-time was calculated from the first negative HIV test to the last negative HIV test or the midpoint of the first positive test and the preceding negative test for those who tested positive [33]. Person-time was also calculated using the first positive HIV test date for those who tested positive; results were only insignificantly changed and results are presented using the midpoint as the time of infection. Participants transferring to other facilities were censored at the last visit date. Cumulative HIV incidence and the cumulative probability of LTFU were estimated using Kaplan-Meier analyses, and the logrank test used to compare exposure categories. Cox’s proportional hazards regression was used to estimate univariable and multivariable hazard ratios (HR) for associations of exposures with incident HIV infection and LTFU. Variables associated with the outcomes were included in multivariable models where their *P*-value in univariable models were ≤ 0.1. The Efron method was used to approximate the exact conditional probability of tied failure times [34]. Proportional hazards assumptions were assessed by comparing Kaplan-Meier observed survival curves and Cox predicted curves for each variable, and statistical testing for nonzero slope of scaled Schoenfeld residuals on functions of time. Model overall goodness-of-fit was assessed by plotting Cox-Snell residuals vs. the Nelson-Aalen cumulative hazard estimator. Data were analysed with Stata version 13.1 (College Station, TX, USA)^(TM)^. The University of Cape Town Human Research Ethics Committee granted ethical approval for the study.

## Results

3480 pregnant women presented for antenatal care during the enrolment period. The following were excluded (S1 Fig): 1583 women known or found to be HIV-infected at the first antenatal visit; 135 who declined consent to participate in the program; 329 who received no follow-up HIV tests (short-term visitors to the area who transferred-out of the program); and 77 whose final HIV test result was < 8 weeks after the initial test. Thus, 1356 women were included in analyses.

At baseline, the median age was 22.5 years (IQR: 19.4–27.0 years) and 30% were adolescents (aged ≤ 19 years) (Table 1). The median gestational age at presentation was 16 weeks (IQR: 12–16 weeks). Almost two-thirds of women requested contraception counselling; 70% required support to access government grants, and 7% requested counselling regarding gender-based violence.

**Table 1:** Baseline characteristics and HIV incidence according to characteristics of pregnant women and their partners in South Africa.

	Total, N (%)	Infections/ 100 PY	Incidence rate per 100 PY (95% CI)	*P*-value*
**Overall**	1356 (100.0)	11/828.3	1.33 (0.74–2.40)	-
**Age at baseline**				0.0303^$^
14–19 years	410 (30.2)	0/248.9	0 (0–1.48)	
20–24 years	488 (36.0)	5/305.4	1.63 (0.68–3.93)	
≥ 25 years	458 (33.8)	6/273.9	2.19 (0.98–4.88)	
**Gestational age at first visit**				
< 14 weeks	427 (31.5)	4/278.2	1.44 (0.54–3.83)	0.853
14–27 weeks	882 (65.0)	7/526.1	1.33 (0.63–2.78)	
≥ 28 weeks	47 (3.5)	0/24.0	0 (0–15.3)	
**Requested contraception counselling?**				0.72
Yes	892 (65.8)	9/631.7	1.42 (0.74–2.73)	
No	464 (34.2)	2/196.5	1.01 (0.25–4.06)	
**Support to access social security grant**				0.748
Support given	954 (70.4)	9/693.7	1.30 (0.67–2.49)	
Support not given	402 (29.7)	2/134.5	1.49 (0.37–5.94)	
**Required gender-based violence counselling or referral**				0.368
No	1264 (93.2)	11/769.5	1.43 (0.79–2.58)	
Yes	92 (6.8)	0/58.7	0 (0–6.2)	
**Initial circumcision status of partner**				0.011
circumcised	625 (46.1)	1/393.0	0.25 (0.04–1.80)	
not circumcised	484 (35.7)	7/303.9	2.30 (1.10–4.83)	
not recorded	247 (18.2)	3/131.4	2.28 (0.73–7.07)	
**Partners HIV status**				<0.0001
HIV uninfected	717 (52.9)	0/544.1	0 (0–0.7)	
Known HIV infected at enrolment	4 (0.3)	1/2.48	40.3 (5.67–286.0)	
Newly diagnosed HIV-infected	5 (0.4)	4/1.81	221.4 (83.1–590)	
not HIV tested/unknown status	630 (46.5)	6/279.9	2.1 (0.96–4.77)	

*P-values estimated by the log-rank test comparing HIV incidence.

^$^log-rank test for trend.

PY-person years.

### HIV incidence

**I**ncluded women received 5289 HIV tests with a median of 4 HIV tests per woman. During 828.3 person-years of follow up, 11 new HIV infections were detected, yielding an HIV incidence rate of 1.33 infections/100 PY (95% CI: 0.74–2.40). Cumulative HIV incidence after 12 months of follow-up was 0.92% (95% CI: 0.51%-1.66%).

Antenatally (i.e. between the first negative antenatal HIV test and delivery), eight new HIV infections were detected during 537 person-years of follow-up, with an antenatal HIV incidence rate of 1.49 infections/100 PY (95% CI: 0.64 to 2.93) and cumulative HIV incidence during pregnancy of 0.68% (95% CI: 0.29%-1.33%). Postnatally, three new cases of HIV were detected over 291 person-years, with an incidence rate of 1.03 infections/100 PY (95% CI: 0.33–3.19).

**Fig 1:**
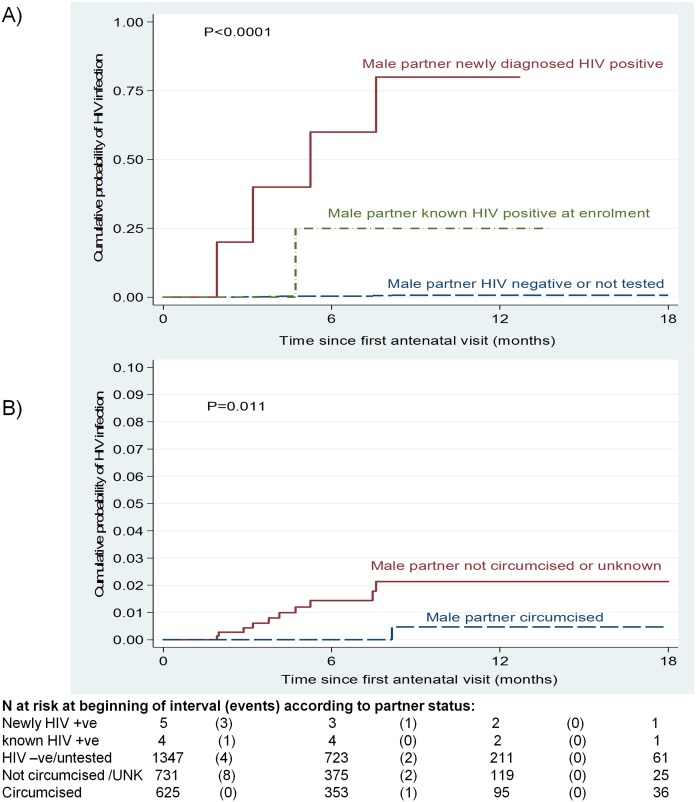
Kaplan-Meier failure estimates of time till HIV infection amongst HIV-negative pregnant women. A) According to male partner’s HIV serostatus, B) According to male partner’s circumcision status.

Table 1 shows HIV incidence according to socio-demographic characteristics of women and their partners. Women with known HIV-infected partners at enrolment had an HIV incidence rate of 40.3 infections/100 PY (95% CI: 5.67–286.0). Women whose partners had newly diagnosed HIV infection had very high HIV incidence, being 221.4 infections/100 PY (95% CI: 83.1–590) with a cumulative probability of acquiring HIV twelve months after the first visit of 80.0% (95% CI: 41.8%-99.1%) (Fig 1A). In contrast, women whose partners were HIV-uninfected or who had unknown HIV serostatus had an incidence rate of 0.73 infections/100 PY (95% CI: 0.33–1.62) and a cumulative probability of acquiring HIV of 0.8% (95% CI: 0.3%-1.8%) twelve months after the first visit (logrank P <0.0001). Amongst women with incident HIV infection (n = 11), 45.5% had HIV-infected male partners and the remaining partners had unknown HIV serostatus.

HIV incidence in women was higher where the male partner was uncircumcised (2.30 infections/100 PY [95% CI: 1.10–4.83] vs. 0.25 infections/100 PY [95 CI: 0.04–1.80] where male partner circumcised; P = 0.011) (Fig 1B).

Table 2 shows univariable and multivariable analyses of factors associated with incident HIV infection (n = 1356). Women with known HIV-infected male partners, adjusted hazard ratio (aHR) = 32.7 (95% CI: 3.8–282.2), and women whose partners were newly diagnosed with HIV infection, aHR = 126.4 (95% CI: 33.8–472.2) had substantially increased HIV acquisition. Women with circumcised partners at baseline had a reduced risk of incident HIV infection, although this did not reach statistical significance at the 95% level in the multivariable model, aHR = 0.22 (95% CI: 0.03–1.86).

**Table 2:** Univariable and multivariable Cox regression of factors associated with incident maternal HIV infection (n = 1356).

	Univariable Hazard Ratio (HR)	Univariable 95% CI for HR	*P*	Multivariable HR	95% CI for multivariable HR	*P*
**Age at baseline**						
< 25 years	1.0 (Ref)	-				
≥ 25 years	2.42	0.73–7.94	0.14	-		
**Gestational age at first visit**						
< 14 weeks	1.0 (Ref)	-				
≥ 14 weeks	0.92	0.26–3.15	0.90	-		
**Required contraception counselling**	1.32	0.28–6.21	0.73	-		
**Received support to access social security grant**	0.77	0.15–3.79	0.75	-		
**Male partner’s initial circumcision status**						
not circumcised or unknown	1.0 (Ref)	-		1.0 (Ref)	-	
circumcised	0.11	0.01–0.86	0.035	0.22	0.03–1.86	0.16
**Male Partner’s HIV status**						
uninfected or not tested	1.0 (Ref)	-		1.0 (Ref)	-	
known HIV-infected	52.4	6.3–438.3	<0.0001	32.7	3.8–282.2	0.001
newly diagnosed HIV infection	202.3	56.9–719.0	<0.0001	126.4	33.8–472.2	<0.0001

### Secondary outcomes

Table 3 lists the secondary study outcomes. Women with incident HIV infection initiated ART on the same day as HIV diagnosis in most cases, and all women with antenatal incident HIV infection initiated ART antenatally. Amongst women with incident HIV infection, MTCT at 6 weeks postpartum was 22.2%.

**Table 3:** Secondary study outcomes evaluating a combination HIV prevention intervention amongst pregnant and postpartum women in South Africa.

	Point estimate	95% Confidence interval (or IQR)
**Antenatal HIV incidence, infections/100 person-years**	1.48	0.64 to 2.92
**Postnatal HIV incidence, infections/100 person-years**	1.03	0.33 to 3.19
**Vertical HIV transmission amongst infants (at age six weeks) amongst women with incidence HIV infection, n/N (%)**	2/9* (22.2%)	2.8% to 60%
**Time since diagnosis until initiating ART amongst women with incident HIV infection, days**	0	IQR: 0–0
**Proportion of women with incident antenatal HIV infection who initiated ART antenatally, n/N (%)**	8/8 (100%)	63.1% to 100%
**Maternal mortality rate, deaths/person-years (deaths/100 person-years)** ^&^ ^#^	1/832.7 (0.12)	0.02 to 0.85
**Cumulative probability of loss to follow-up 12 months after the first antenatal visit, %**^#^	10.8%	9.1% to 12.8%
**Proportion of eligible male partners received HTC with recorded test results, n/N (%)**	722/1352 (53.4%)	50.7% to 56.1%
**Proportion of male partners who received HTC with newly diagnosed HIV infection, n/N (%)**	5/722 (0.7%)	0.2% to 1.6%
**Proportion of newly diagnosed HIV-infected male partners linked to HIV care facilities, n/N (%)**	5/5 (100%)	47.8% to 100.0%
**Time from HIV diagnosis till ART initiation amongst males partners eligible to initiate ART, days**	7	-
Proportion of eligible male partners referred for VMMC, n/N (%)**	476/722 (65.9%)	68.9% to 76.1%

* Two women tested HIV positive more than six weeks after birth, thus their infants did not receive HIV testing at the six week immunization visit.

^#^ From Kaplan-Meier analyses.

** Eligible males were uncircumcised males (or who had unknown circumcision status) who tested HIV negative or who had unknown HIV status.

^&^ Deaths during pregnancy or until 42 days of termination of pregnancy related to or aggravated by the pregnancy

ART-antiretroviral treatment; IQR-interquartile range; VMMC-voluntary male medical circumcision; HTC-HIV testing and counselling.

One (0.07%) maternal death was recorded, with a maternal mortality rate of 0.12 deaths/100 PY (95% CI: 0.02 to 0.85). During the study period, 122 (9.0%) women become LTFU. The cumulative probability of LTFU twelve months after the first antenatal visit was 10.8% (95% CI: 9.1%-12.8%). Table 4 shows the multivariable model of factors associated with LTFU (n = 1356; 122 events). Women who first attended antenatal facilities in late pregnancy and women aged ≥ 25 years had increased risks of LTFU. Women who received support to access social security grants, women whose partners received HTC, and women who received family planning counselling had reduced LTFU.

**Table 4:** Factors associated with loss to follow-up amongst pregnant and postpartum women enrolled in a combination HIV prevention intervention in South Africa.

	Univariable Hazard Ratio (HR)	Univariable 95% CI for HR	*P*	Multivariable HR	95% CI for multivariable HR	*P*
**Age at baseline**						
< 20 years	1.0 (Ref)	-		1.0 (Ref)	-	
20–24 years	1.11	0.69–1.78	0.66	1.08	0.67–1.73	0.75
≥ 25 years	1.58	1.00–2.47	0.047	1.57	1.00–2.47	0.048
**Gestational age at first visit**						
< 14 weeks	1.0 (Ref)	-		1.0 (Ref)	-	
14–27 weeks	1.14	0.77–1.69	0.50	1.63	1.07–2.42	0.015
≥ 28 weeks	1.99	0.83–4.71	0.10	3.63	1.52–8.70	0.004
**Required gender-based violence counselling or referral**	1.64	0.93–2.92	0.090	1.52	0.78–2.96	0.21
**Received contraception counselling**	0.22	0.16–0.32	<0.001	0.58	0.35–0.97	0.036
**Received support to access social security grant**	0.14	0.09–0.20	<0.001	0.31	0.18–0.53	<0.001
**Male partner received HTC**						
No	1.0 (Ref)	-			1.0 (Ref)	-
Yes	0.16	0.10–0.24	<0.001	0.30	0.18–0.51	<0.001

HTC-HIV testing and counselling.

Amongst male partners, 722 (53.4%) received HTC and had a recorded HIV test result, of whom 60.0% received home-based HTC. Five (0.7%) partners were newly diagnosed with HIV infection. Amongst those women who acquired HIV and who had male partners with newly diagnosed HIV infection (n = 4), all the partners first accepted HTC only after the women tested HIV-positive (median 4.5 days). All the newly diagnosed HIV-infected male partners were successfully linked with HIV care facilities; however, only 1/5 (20%) initiated ART (seven days after HIV diagnosis), as the remainder had CD4 cell counts greater than 500 cells/μL and were not eligible to initiate ART according to prevailing South African ART guidelines. Amongst partners with recorded HIV serostatus and recorded baseline circumcision status, HIV prevalence was considerably higher in uncircumcised males (3.3%) vs. 0.0% in circumcised males (P <0.0001). Amongst partners eligible for VMMC, 65.9% accepted referral for VMMC.

## Discussion

In a high HIV incidence area of South Africa, HIV incidence was found to be low amongst pregnant and postpartum women who received a community-based combination individual and couple’s HIV prevention intervention. Previous studies have found HIV incidence amongst nonpregnant female adolescents in the same district to be 17.2 infections/100 PY [28]; 10.7 infections/100 PY during pregnancy (all ages) in South Africa [6]; and 16.8 infections/100 PY during pregnancy in neighbouring Swaziland [35]. A recent systematic review and meta-analysis of HIV incidence during pregnancy/postpartum in Southern Africa indicated HIV incidence of 4.8 infections/100 PY (95% CI: 3.5–6.4) [11]. Recent South African data from a national survey estimated cumulative HIV incidence during pregnancy to be 3.3% (95% CI: 2.8%-3.8%) [14]. The maternal HIV incidence rate measured in our study thus represents a decrease of 73%–86% compared to previously published Southern African studies.

One of South Africa’s strategic health objectives is to reduce HIV incidence by 50% by the end of 2016 [36]. However, to decrease HIV incidence and prevalence over a 50-year time-scale, it has recently been shown that substantial scale-up of combination HIV prevention programs will be required [37]. Community health workers have been drafted as a priority workforce in South Africa’s approach to re-engineering primary healthcare [25]. This community-based intervention shows promise in reducing high HIV incidence amongst pregnant and postpartum women.

MTCT amongst women acquiring HIV during pregnancy was high, similar to previous studies [12,13], and is due to high maternal HIV viral load and reduced transfer of protective immunity to the child during acute maternal infection [38].

Male involvement and uptake of partner HTC in antenatal settings in sub-Saharan African is low [39–41]. Partner HTC uptake in this intervention was considerably higher than in a previous South African trial (11%-32%) [42]. Most previous studies investigating incident HIV infection during pregnancy/lactation did not attempt to record male partner’s HIV serostatus [6,10,35,43,44]. One Kenyan study recorded partner’s HIV status as reported by women [9]. Our study is unusual in that it attempted to directly measure partner’s HIV serostatus; and found that almost half of incident infections in women were associated with the main partner being HIV-infected. It is notable that amongst woman who acquired HIV and whose partners tested HIV-positive, partners first accepted HTC only after the woman seroconverted. The serostatus of the partner was thus initially unknown, and the couple may not have taken precautions to minimize transmission. Prioritizing HTC of partners (and initiating ART if required) as early as possible during pregnancy is thus important to detect serodiscordant couples and to minimize transmission risk.

Circumcision does not decrease the risk of HIV transmission from HIV-infected men to uninfected women [45]. The protective effect of partner circumcision in our study is likely an indirect effect related to lower HIV prevalence amongst circumcised partners who had unknown HIV status, as amongst males with known HIV status, uncircumcised males had considerably higher HIV prevalence.

Concerns and uncertainty about the safety of tenofovir disoproxil fumarate (TDF)-based PrEP during pregnancy and lactation have prevented large-scale PrEP implementation amongst women of child-bearing age. Indeed, current South African public sector HIV guidelines consider PrEP use for commercial sex workers only [46,47], while private sector guidelines consider PrEP contraindicated during pregnancy and breastfeeding and do not address the issue of PrEP for HIV-uninfected pregnant and breastfeeding women within serodiscordant couples [18]. Given the efficacy of PrEP in preventing HIV acquisition amongst adherent women (up to 75% risk reduction in heterosexual African couples) [48], and balanced with potential concerns of the effect of PrEP on the fetus/infant, it has recently been suggested that studies should identify subgroups of pregnant/postpartum women at high risk of acquiring HIV, and limit provision of PrEP to these subgroups [9,49]. Notably, studies involving breastfeeding HIV-infected women and their infants showed very low exposure to TDF from breast milk [50,51]. Also, duration of in-utero exposure to TDF was not associated with infant early linear growth [52]. There are few studies of TDF in HIV-uninfected women. However, data from hepatitis B virus (HBV) mono-infected pregnant women showed foetal/infant exposure to TDF was not associated with any adverse outcomes, and TDF reduced perinatal HBV transmission [53–55]. Data from two PrEP studies in HIV-uninfected women are similarly reassuring [19,56]. Given the limited available safety data, ongoing surveillance is needed, but the benefits of PrEP use for pregnant/lactating women at high risk of HIV acquisition (and associated increased risk of MTCT) appears to far outweigh the potential risks of foetal, infant and maternal TDF exposure [57]. Therefore, strong consideration should be given to revising South African guidelines to include initiation and maintenance of PrEP for HIV-uninfected women within serodiscordant couples during pregnancy and breastfeeding. Recent cost-effectiveness modelling has shown that provision of PrEP to pregnant/breastfeeding women in sub-Saharan Africa would likely be cost-effective [49].

Challenges to the program included women not always being available for home visits or clinic groups because of work commitments; broaching issues regarding age-disparate and concurrent sexual relationships during individual counselling; and inconsistent uptake of HTC amongst men.

The strengths of the study include that prospective cohort data were collected that enabled detection of incident HIV throughout pregnancy and postpartum. The routine urban setting with high HIV incidence indicates that results would likely be generalizable to other high HIV incidence Southern African settings. LTFU was reasonable, considering that LTFU of pregnant women from health programs in Southern Africa may be substantial [58,59].

The study limitations include the lack of a comparison cohort who did not receive the intervention to directly measure the effect of the intervention. Due to the data’s routine nature, a relatively small number of potential predictors of HIV acquisition were available for analysis. Although the few incident HIV infections was encouraging, low case numbers resulted in imprecise effect measures of factors associated with incident HIV infection. Partner HIV testing frequently post-dated that of the female partner due to slower uptake of male HIV testing, thus for those partners who tested HIV-positive at initial testing, we could not distinguish prevalent male partner HIV infection at baseline (i.e. the pregnant women’s first antenatal clinic visit) from incident male HIV infection during the woman’s follow-up. The proportion of referred men who received VMMC was also unavailable in the routine dataset.

In conclusion, low HIV incidence amongst pregnant and postpartum women in a high HIV incidence setting was observed amongst women who received a community-based combination HIV prevention intervention for women and couples. Incident HIV cases were highly associated with serodiscordant couples and new HIV diagnoses in male partners. Expanded roll-out of combination HIV prevention interventions incorporating home-based couples HTC for pregnant women and partners in high HIV prevalence settings should be considered, attempting early uptake of HTC amongst males and referral for VMMC or ART initiation as indicated. Together with ART for HIV-infected partners, initiating PrEP for HIV-uninfected pregnant and breastfeeding women within serodiscordant couples should be considered to decrease both maternal HIV incidence and pediatric HIV. Increased frequency of HIV testing of pregnant women (4–6 weekly) should be considered, and further research is needed regarding innovative and feasible strategies to enhance early HTC uptake for partners.

## Supporting information

S1 Fig Flow chart of women included and excluded from analyses. (PDF)
