# Supplementary figures and images for: Low HIV incidence in pregnant and postpartum women receiving a community-based combination HIV prevention intervention in a high HIV incidence setting in South Africa
Source: PLoS One. 2017 Jul 27;12(7):e0181691. doi: 10.1371/journal.pone.0181691 (PMC5531482; doi:10.1371/journal.pone.0181691)

## Supplemental Digital Content 1: Flow chart of women included and excluded from analyses

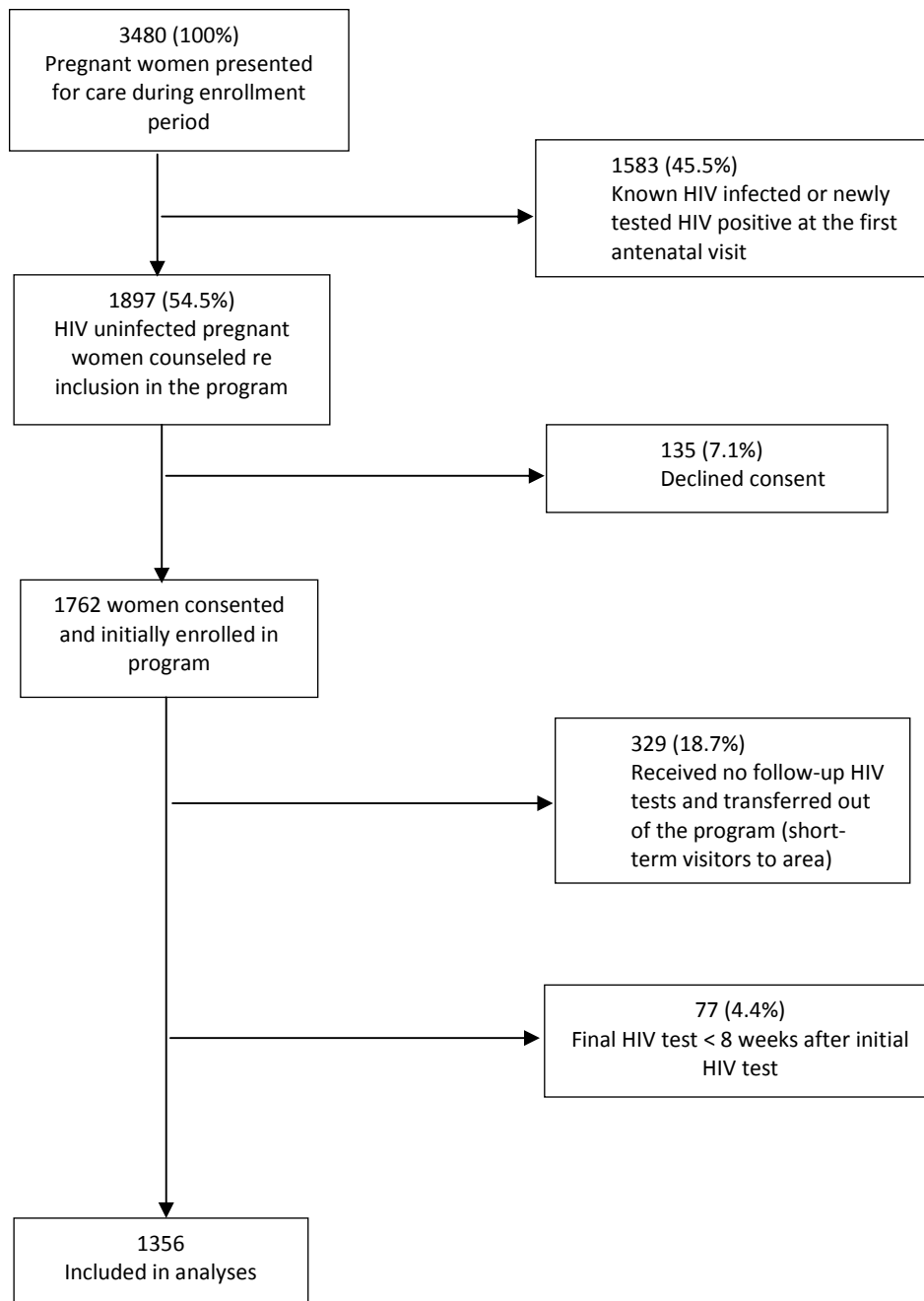

Supplement: S1 Fig — (PDF) [file pone.0181691.s001.pdf]
